# Chemical Multiverse: An Expanded View of Chemical Space

**DOI:** 10.1002/minf.202200116

**Published:** 2022-08-23

**Authors:** José L. Medina‐Franco, Ana L. Chávez‐Hernández, Edgar López‐López, Fernanda I. Saldívar‐González

**Affiliations:** ^1^ DIFACQUIM research group, Department of Pharmacy, School of Chemistry National Autonomous University of Mexico Mexico City 04510 Mexico; ^2^ Department of Pharmacology Center for Research and Advanced Studies of the National Polytechnic Institute (CINVESTAV) Mexico City 07360 Mexico

**Keywords:** chemical multiverse, chemical space, drug discovery, machine learning, molecular representation, structure-property relationships, ultra-large chemical library, visualization

## Abstract

Technological advances and practical applications of the chemical space concept in drug discovery, natural product research, and other research areas have attracted the scientific community‘s attention. The large‐ and ultra‐large chemical spaces are associated with the significant increase in the number of compounds that can potentially be made and exist and the increasing number of experimental and calculated descriptors, that are emerging that encode the molecular structure and/or property aspects of the molecules. Due to the importance and continued evolution of compound libraries, herein, we discuss definitions proposed in the literature for chemical space and emphasize the convenience, discussed in the literature to use complementary descriptors to obtain a comprehensive view of the chemical space of compound data sets. In this regard, we introduce the term *chemical multiverse* to refer to the comprehensive analysis of compound data sets through several chemical spaces, each defined by a different set of chemical representations. The chemical multiverse is contrasted with a related idea: consensus chemical space.

## Graphical abstract

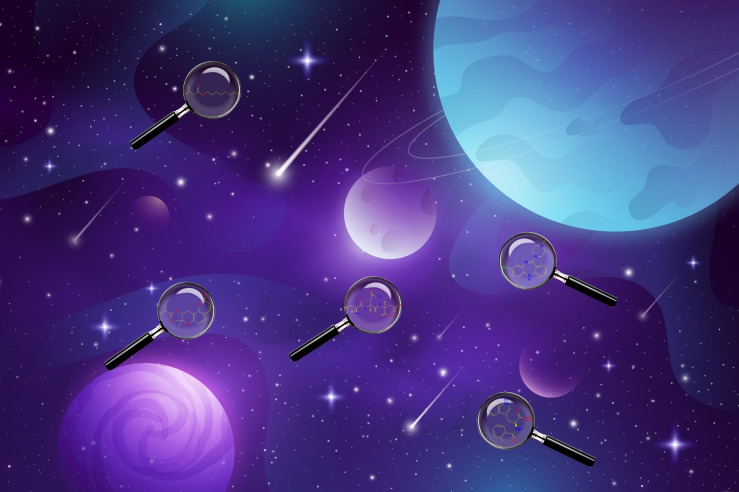

## 1 Introduction

The concept of “chemical universe,” “chemical space,” or “chemical compound space” is associated with a set of all possible molecules described by a multi‐dimensional space that represents their functional and structural properties and the relationship of the molecules to each other.[^1^, ^2^] Although the type of molecules could be any, the chemical space has been typically studied quantitatively and qualitatively with small organic compounds. Practical applications in drug discovery approaches include the study of epigenetic‐focused compounds,[^3^, ^4^] covalent protein kinase inhibitors,^[5]^ human immunodeficiency virus (HIV) protease inhibitors,^[6]^ natural products (NPs),[^7^, ^8^] and novel compounds from combining fragments or scaffolds, e. g., pseudo‐NPs.^[9]^ Also, there are reported applications of the chemical space concept to food and flavor chemicals,^[10]^ peptides,^[11]^ and metal‐containing molecules,^[12]^ and virtual and on‐demand libraries.^[13]^

In theory, the multi‐dimensional space can be formed by two (or even one) dimensions, e. g., a single or two descriptors that encode a specific set of structural or functional properties. However, depending on the project‘s goals, the dimensions are typically more than three. Eventually, it could contain hundreds or a few thousands of descriptors, for instance, when using structural fingerprints. Many descriptors demand the implementation of dimensionality‐reduction techniques to generate two (2D) – or three‐dimensional (3D) visual representations of the multi‐dimensional descriptor space. Reduction methods such as t‐distributed stochastic neighbor embedding (t‐SNE),^[14]^ principal component analysis (PCA),^[15]^ self‐organized maps (SOMs),^[16]^ generative topographic mapping (GTM),[^17^, ^18^, ^19^] and chemical space networks[^20^, ^21^] currently are the most frequently used, but there are others. Visual representation of the chemical space has been the focus of several research projects, as further discussed below.

For several decades, the chemical space concept has been of interest in several areas of chemistry, emphasizing drug discovery. With the rapid advances in machine learning and *de novo* design and the number of chemical compounds that exist or could be made, the community has a significant interest in enumerating large‐ and ultra‐large chemical libraries containing billions of chemical structures.^[22]^ Hence, there is an increased interest in studying the huge chemical libraries under the chemical space concept, e. g., systematic and consistent description with novel and existing chemical descriptors, visual representation of the chemical space of libraries with millions of compounds, and several other analyses that can be done based on the multi‐dimensional chemical space (examples of the latter are diversity analysis, similarity‐based virtual screening, property, and biological activity prediction). For instance, Schmidt et al. and Bellmann et al. recently explored the chemical space of make‐on‐demand libraries.[^13^, ^23^] Zabolotna et al. reported the implementation of GTM to efficiently navigate the chemical space of the entire ZINC library of purchasable compounds, relative to the biologically relevant ChEMBL compounds.^[19]^

There are several reviews focused on chemical space. These reviews, summarized in Table 1, cover applications to drug discovery and NPs research, progress on visualization methods, and approaches to study the chemical space of small organic compounds, emphasizing the application of public resources. However, the review or research papers using the concept of chemical space often have a narrow or very focused view of the space. For instance, they usually refer to organic (typically small) molecules.^[24]^

**Table 1:** Selected review papers focused on the chemical space of different types of compounds.^a^

Review title	Focus of the review	Ref.
Visualization of the chemical space in drug discovery.	Summarized chemical space visualization, several programs to visualize chemical space for chemical compounds from natural products, drug molecules, and combinatorial libraries, and their application in drug discovery.	^[25]^
Exploring chemical space for drug discovery using the chemical universe database.	Progress on the authors in searching for bioactive ligands by enumeration and virtual screening of the unknown chemical space of small molecules. The review covers the application of these libraries to the search for NMDA‐receptor analogous.	^[26]^
The chemical space project.	Development of chemical universe databases (GDB), a project to enumerate all possible molecules to generate an unbiased insight into the entire chemical space, taking only simple chemical stability and synthetic feasibility criteria.	^[27]^
Progress in visual representation of chemical space.	Chemical space concepts. Advances in methods for visualization of chemical space, and older but overlooked methods.	^[28]^
Reaching for the bright StARs in chemical space.	Visualization methods to explore the chemical space aiming at reaching insightful structure‐activity relationships.	^[29]^
Chemoinformatics in natural product‐based drug discovery.	General review of chemoinformatics applied to natural products. It includes analysis, visualization, and navigation of their chemical space.	^[30]^
Defining and exploring chemical spaces.	Overview of algorithmic approaches to defining and exploring chemical spaces that have the potential to operationalize the process of molecular discovery.	^[31]^
Ab initio machine learning in chemical compound space.	Machine learning studies aimed at sampling exhaustively the chemical compound space. The review covers novel molecules in general (non‐only small organic molecules) and materials.	^[32]^
Progress on open chemoinformatic tools for expanding and exploring the chemical space.	Recent progress on chemoinformatic tools to expand and characterize the chemical space of compound data sets employing various types of molecular representations, generate visual representations of the chemical space and analyze the SAR of data sets.	^[33]^
Using deep neural networks to explore chemical space.	Common deep learning methods to explore the chemical space. The review discusses the selection of molecular representation, training for focused chemical space exploration, and considerations for assessing and validating the chemical space coverage.	^[34]^
Approaches for enhancing the analysis of chemical space for drug discovery.	The current state of chemical space in drug design and discovery. Topics discussed: advances for efficient navigation in chemical space, the use of this concept in assessing the diversity of different data sets, exploring SAR for one or multiple endpoints, and compound library design.	^[35]^

^a^ In chronological order of publication.

This manuscript's goal is to review definitions proposed in the literature for chemical space, emphasizing the approaches to generate a consensus view of the chemical space. Building upon the developments of others and our group on chemical space, we also propose the term *chemical multiverse* highlighting the convenience of employing multiple descriptors for a comprehensive assessment of the chemical space. After this brief introduction, we discuss the current definitions of chemical space. Section 3 introduces the term of chemical multiverse and compares it with the notion of consensus chemical space. In Section 4 we review the applications discussed in the literature of multiple descriptors to analyze the chemical space of compound data sets. In other words, we present case studies discussed in the literature showing the general applicability of chemical multiverses in diversity analysis, virtual screening, and structure‐activity relationships, among other applications.

## 2 Current Views of Chemical Space

There are several definitions of chemical space proposed in the literature. Table 2 summarizes examples.

**Table 2:** Examples of definitions of chemical space concepts, as proposed in the literature.^a^

Author(s)	Chemical space definitions	Ref.
Dobson	“All possible small organic molecules, including those present in biological systems”.	^[36]^
Lipinski and Hopkins	“Chemical space can be viewed as being analogous to cosmological universe in its vastness, with chemical compounds populating space instead of stars”.	^[37]^
Reymond, et al.	“Ensemble of all known and possible molecules described by their chemical properties”.	^[38]^
Varnek and Baskin	“The ensemble of graphs or descriptor vectors forms a chemical space in which some relations between the objects must be defined”.	^[39]^
von Lilienfeld, et al.	“The combinatorial set of all compounds that can be isolated and constructed from possible combinations and configurations of N_1_ atoms and N_e_ electrons in real space”.	^[40]^
Virshup et al.	“An M‐dimensional cartesian space in which compounds are located by a set of M physicochemical and/or chemoinformatic descriptors”.	^[2]^
Vogt	“Comprehensive collection of all possible small molecules under some reasonable restrictions considering size and composition”.	^[41]^
Huang and Lilienfeld	“Chemical compound space is the set of all theoretically conceivable combinations of chemical elements and (meta‐)stable geometries that make up matter”.	^[32]^

^a^ In chronological order of publication.

**Figure 1:**
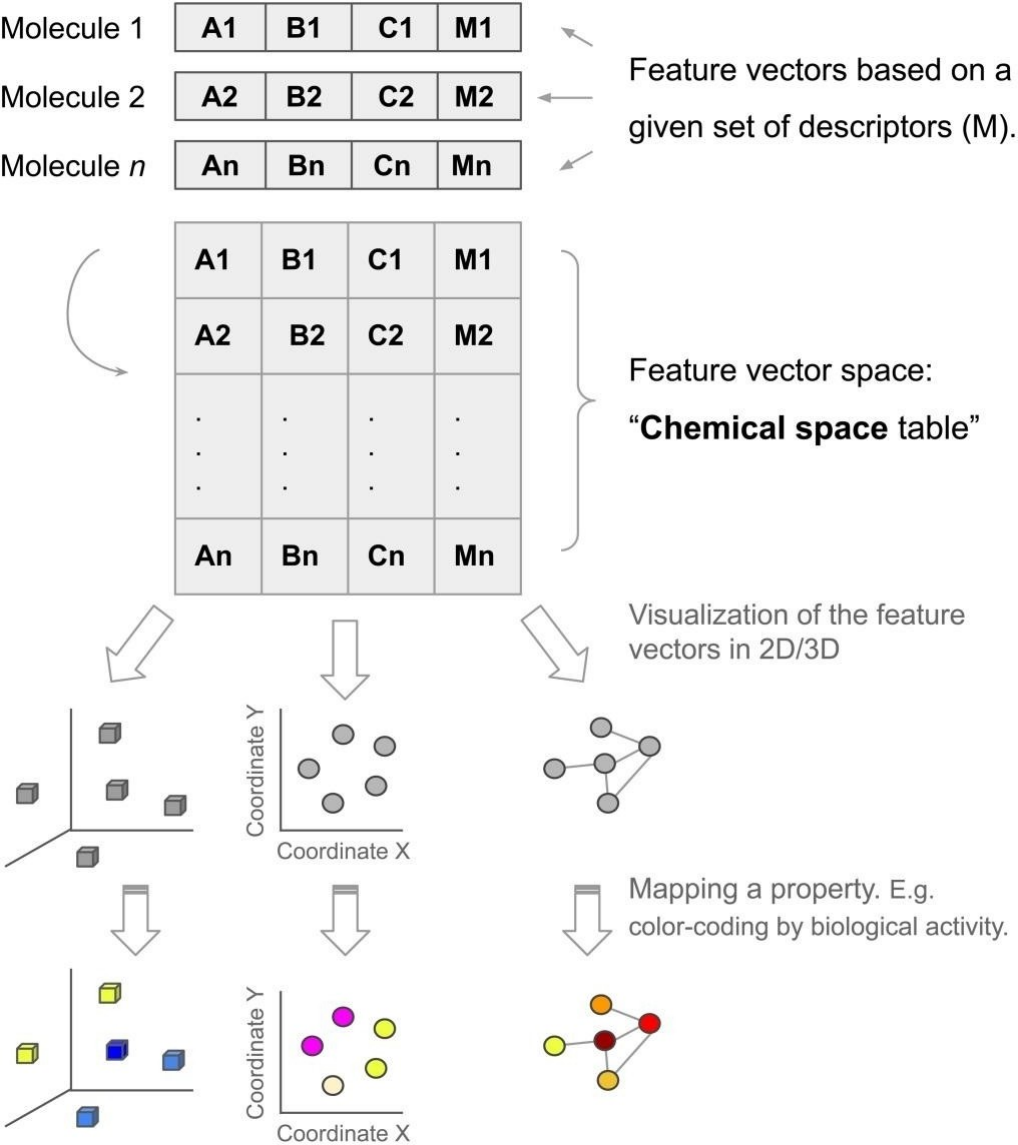
Schematic view of the chemical space. Each molecule in the compound data set is represented by *M* descriptors that lead to a feature vector space. The group of *n* molecules represented with *M* descriptors form a “chemical space table” that can be represented using different visualization methods. Mapping a property (e. g., biological activity) to the “chemical space table” or the visual representation gives rise to a chemogenomic space that is the basis to do structure‐property (activity) relationships.

As reviewed in Table 2, several definitions conceptualize the chemical space as a chemical descriptor vector space set by the numerical vector D encoding property or molecular structure aspects as elements of the descriptor vector D.^[35]^ Based on this notion, Figure 1 shows the chemical space concept like “M‐multidimensional cartesian space,” aka a “chemical space table.” Rows represent the number of (n) molecules, and the columns (M) are the number of descriptors or features that encode each molecule. The length of descriptor sets corresponds to the number of dimensions defining the chemical space itself.

## 3 Chemical Multiverse: General Concept

The chemical space concept implies that a given set of *n* molecules represented with different descriptors would lead to distinct chemical universes e. g., each one is “descriptor universe”. Varnek and Baskin have pointed out that “unlike real physical space, a chemical space is not unique: each ensemble of graphs and descriptors defines its own chemical space.”^[39]^ It also follows that molecules with very different chemical nature, for instance, organic small‐molecules, e. g., lead‐ and drug‐like; peptides; metal‐containing compounds, macromolecules, biologics, etc., yield divergent chemical spaces, this be their own nature of the descriptors required to represent the compounds. In addition, the number and type of chemical and biological‐related descriptors available from experimental data or computational calculations, e. g., quantum mechanics,^[32]^ are also increasing, yielding the chance to augment the number of possible valid chemical spaces.

In physics, Everett's multiverse^[42]^ is “a hypothetical collection of potentially diverse observable universes, each of which would comprise everything that is experimentally accessible by a connected community of observers.”^[43]^ In other words, the multiverse “is a hypothetical group of multiple universes,” and regions in the universe detached from one another exhibit distinct properties.^[44]^

By rough analogy with the cosmic multiverse, here we introduce the term *chemical multiverse* as the group of numerical vectors that describe it differently from the same set of molecules. In other words, a chemical multiverse is a group of multiple chemical spaces, each one defined by a given set of descriptors e. g., a group of “descriptor universes”. Furthermore, and maintaining the analogy with the cosmology megaverse, a “chemical megaverse” is the collection of chemical multiverses. This would be given by the several different sets of descriptors that can be used to define a chemical space (Figure 1). As discussed in previous papers (Table 2), different chemical space representations lead to other spaces, and relationships between chemical compounds could be maintained or not.^[20]^

**Figure 2:**
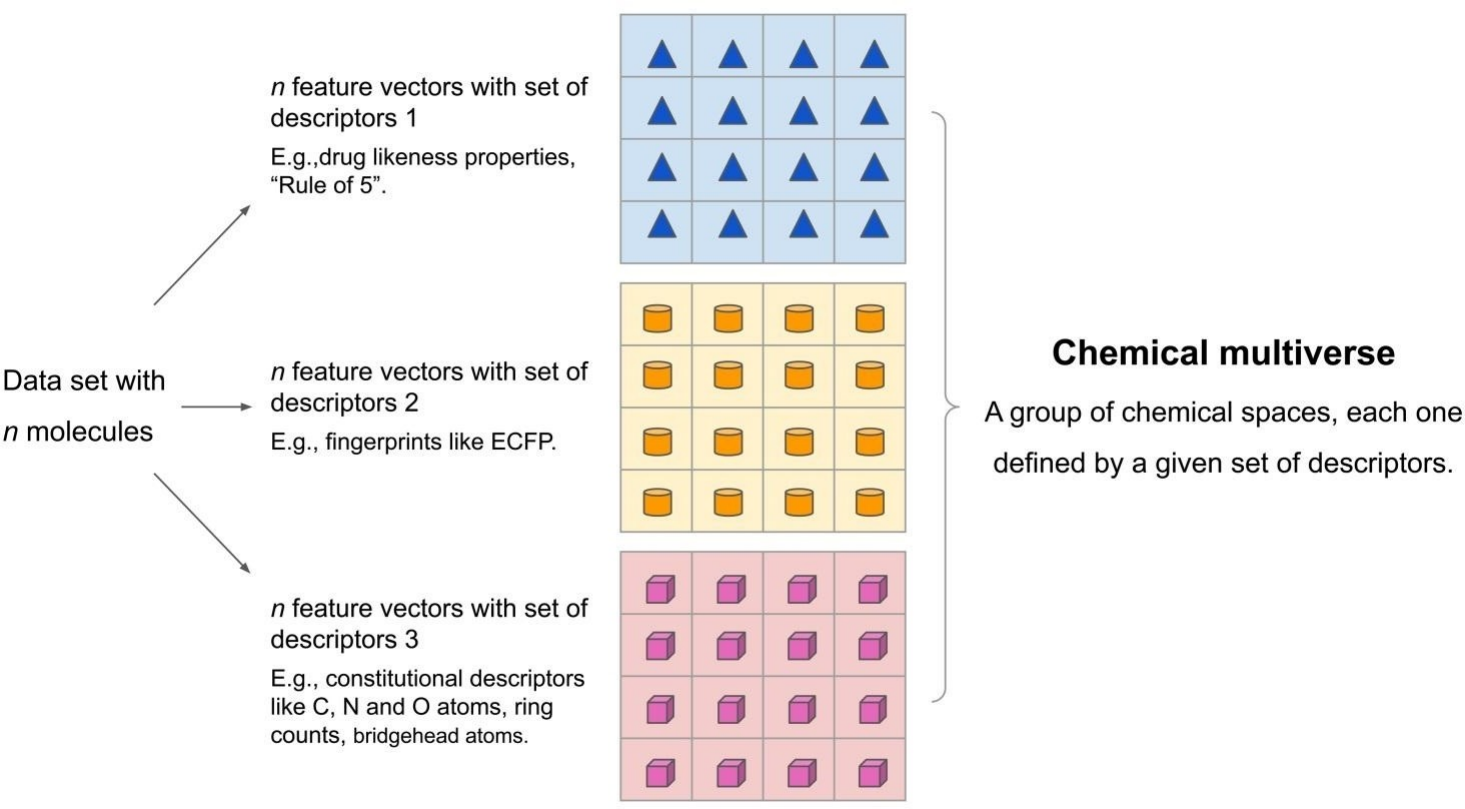
Schematic and general representation of the concept of the chemical multiverse. The chemical multiverse of the same data set with *n* molecules would be composed of several (shown in figure three for illustrative purpose) alternative chemical spaces, each one defined by a different set of descriptors. The geometric figures represent the encoding of the structures using different descriptors: e. g., drug‐likeness properties (blue triangles), fingerprints (orange cylinders), and constitutional descriptors (such as ring counts, carbon, nitrogen, oxygen, and bridgehead atoms) (pink cubes). Depending on the study‘s goals, a chemical multiverse could contain as many chemical spaces as needed. Each chemical space (in the middle section: “chemical space tables”) could be subject to different 2D/3D visual representations of the chemical space.

**Figure 3:**
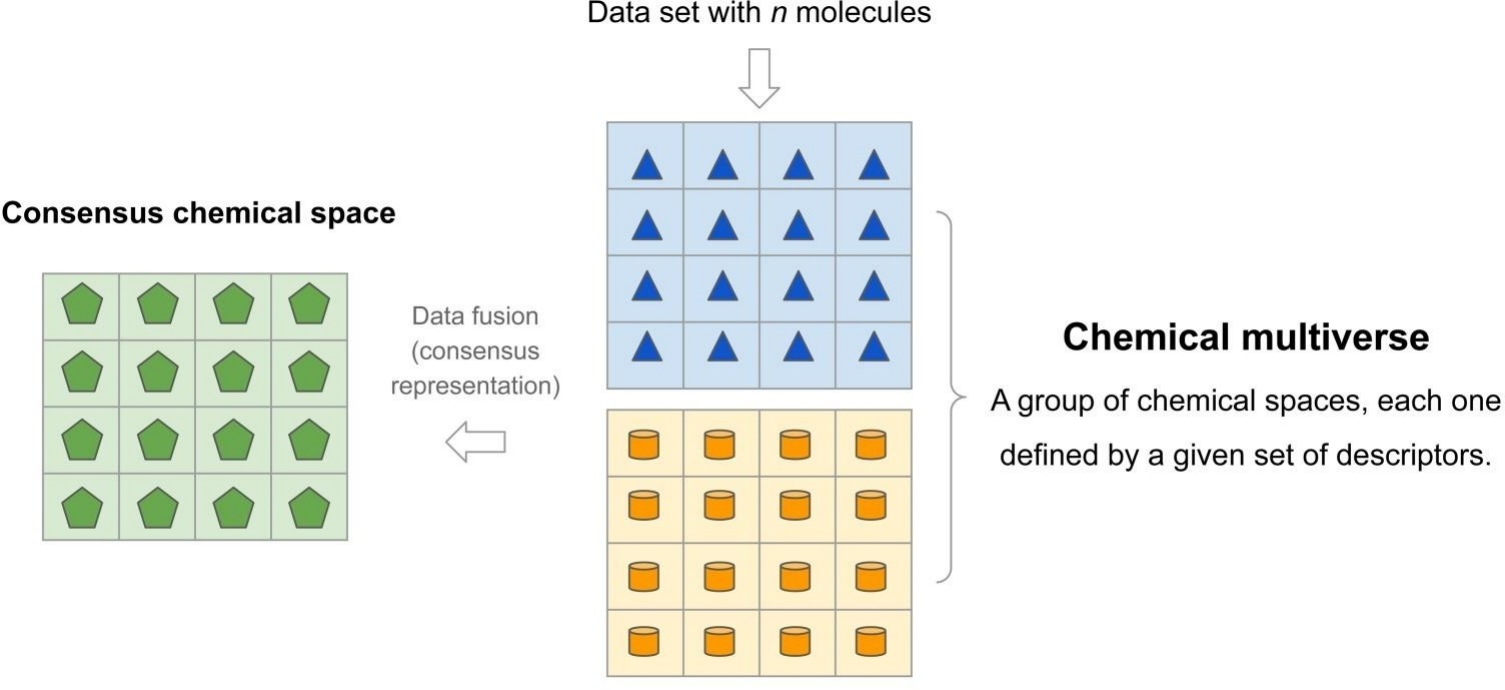
General comparison of a chemical multiverse with consensus chemical space. While a chemical multiverse of a compound data set is a set of alternative chemical spaces (shown in figure only two, for illustration purposes), a consensus chemical space is the combination of the alternative chemical spaces to yield one single chemical space that is the result of data fusion or combination of the descriptors. The grids with blue triangles and orange cylinders encode two different chemical spaces described by, for instance, drug‐likeness and ECFP descriptors, respectively. In this schematic example, the green hexagons represent the fusion or combination of the two descriptors to lead to a new but single consensus chemical space.

The concept of the *chemical multiverse* is schematically shown in Figure 2. Three grids (“chemical space tables”) encode different chemical spaces, each described by a different set of descriptors. For schematic and illustrative purposes, the blue triangles encode a chemical multiverse defined by Lipinksi's “Rule‐of‐five” that displays favorable pharmacokinetic properties in terms of absorption and distribution. The orange cylinders represent a chemical space described by molecular fingerprints as Extended Connectivity Fingerprints (ECFP). The pink cubes encode the chemical space defined by constitutional descriptors like carbon, nitrogen, and oxygen atoms, ring counts, and bridgehead atoms. Of course, any other set of descriptors can be used. All three chemical spaces in Figure 3 comprise the chemical multiverse of the data set with *n* atoms. Again, the chemical multiverse could be formed by more than three chemical spaces, depending on how many different sets of descriptors need to be used to meet the study's goals. Section 4 presents case studies, most of them published in the literature, of chemical multiverses using real data sets (albeit the name “chemical multiverse” has not been used in previous publications).

The importance of the chemical multiverse concept relies on the fact that a comprehensive view of the chemical space of a data set should be given by several representations, as opposed to a single one. This is because a single set of descriptors is limited and does not capture all aspects of the chemical structures. The need to consider multiple descriptors for chemoinformatics applications has been broadly recognized, for instance, in similarity searching^[45]^ and diversity analysis.^[46]^ A case in point is the so‐called consensus diversity plots that consider at least four types of molecular representation to gain a more comprehensive view of the “global” or total diversity of compound data sets in comparative studies. In consensus diversity plots, types of representation such as scaffolds, structural fingerprints, physicochemical properties, and metrics associated with structural complexity have been employed.[^46^, ^47^]

### 3.1 Chemical Multiverse vs. Consensus Chemical Space

The consensus chemical space can be conceptualized as the result of the fusion or combination of the different descriptors into one. Thus, it is highly associated with the concept of data fusion.^[48]^ Under this concept, the consensus chemical space is dependent on the approach to combining the descriptors of the molecules or making hybrid representations.^[49]^ A consensus chemical space can also be seen as the combination of similarity metrics, also reminiscent of data fusion, to generate a unified representation of the chemical space. In contrast, the chemical multiverse, as introduced in the previous section, does not require a fusion of a combination of descriptors for each molecule: the chemical multiverse is composed of a group of alternative representations of the compounds (e. g., using different fingerprints, properties, or descriptors for a given dataset). Figure 3 shows the difference and relationship between the chemical multiverse and consensus chemical space of a given set of *n* molecules.

While the consensus chemical space combines into one several possible universes created by different sets of descriptions, the chemical multiverse herein is conceptualized as a set of parallel universes. Thus, the chemical multiverse could be a better option to handle the chemical spaces because each one will provide information associated with the particular descriptor used and, therefore, be easier to interpret. In contrast, in a consensus representation, the result could be hard to interpret due to combining several representations into one.

Analogous to the molecular representations,^[50]^ there is no unique and global chemical universe. Whereas a consensus chemical space is an attempt to generate “a single chemical universe,” the relevant information from a set of descriptors can be lost due to combining the chemical spaces, as depicted in Figure 3.

## 4 Examples of Chemical Multiverses

This section reviews case studies of chemical multiverses applied to different data sets of molecules (though the term “chemical multiverse” has not been used before). The chemical multiverses could be analyzed, navigated, and compared, considering the full‐dimension space, similar to individual chemical spaces.^[30]^ Even though the chemical space is typically associated with small organic molecules for drug discovery applications (see exemplary proposed definitions of chemical space in Table 2), we emphasize that the chemical space and chemical multiverse could involve other types of chemical compounds. Table 3 summarizes recent studies of the chemical space using multiple structure representations for different aims. The studies are briefly discussed below.

**Table 3:** Examples of studies of chemical spaces of compound data sets using multiple representations.^a^

Study aims	Data sets and molecular representations and descriptors	Ref.
Structure‐activity relationships	2D representations of the chemical space combined with biological activity using 11 2D and 3D structural representations. The therein generated activity landscapes were used to identify consensus activity cliffs.	^[51]^
Diversity analysis	3D projections of PCA‐based chemical spaces generated from a set of 2250 compounds obtained from nine datasets of 250 compounds each using four fingerprints (atom pairs, MACCS keys, TGD, and piDAPH4) and the Tanimoto coefficient. The visualizations of the chemical space were employed to analyze the diversity of the data sets.	^[52]^
Virtual screening	Eight universal GTMs each generated with different descriptor vectors (In SIlico design and Data Analysis – ISIDA descriptors), each encoding distinct structural features, were employed as support for predictive classification landscapes.	^[17]^
Compound library design	Four PCA plots of molecular quantum number fingerprints to assess the quality of the training process in generative models.	^[53]^
Chemical space navigation	Visualization and navigation through the chemical space of natural products and natural products‐like molecules considering chemotype distribution, physicochemical properties, biological activity, and commercial availability.	^[8]^
Diversity analysis and compound(s) selection	CLNs of 19 chemical libraries used in drug discovery and natural products research were generated using four fingerprints (MACCS keys, RDKit, and ECFP4), and the extended Tanimoto index. CLNs were used to compare the diversity of the data sets.	^[54]^
Diversity analysis	16 comparative ChEMBL vs. purchasable building blocks (PBB) landscapes using GTMs and ISIDA fragment descriptors. GTMs allowed the identification of the most represented and underrepresented classes of PBBs.	^[55]^

^a^ In chronological order of publication.

It has been noted that different similarity measures generate different chemical spaces. For instance, in 2014 Medina‐Franco and Maggiora illustrated the diversity of nine compound data sets as they are projected into 3D obtained from pairwise structure similarity computed with the Tanimoto coefficient and four fingerprints of different designs.^[52]^ For comparison, the authors generated a single visualization of the chemical space obtained by the mean fusion of the four different sets of similarity values (an example of representation of a consensus chemical space). From the visual representation of the chemical multiverse and consensus chemical space was concluded that, albeit graphically, those compound neighborhoods will not remain invariant to changes in molecular representation.

In separate work, Casciuc et al. showed the complementarity of seven GTMs based on a different descriptor space to classify actives and inactive compounds in ChEMBL. In that work, the authors also compared the performance of virtual screening of the complementarity of different maps vs. a consensus map concluding that “while any single universal map has moderate predictive power, the combination of complementary maps lead to a more robust consensus effect in virtual screening.^[17]^ Also using GTMs, Zabolotna et al. presented “NP‐Navigator”, a freely available intuitive online tool for visualization and navigation through the chemical space of NPs and NP‐like molecules.^[8]^ The different representations generated in NP‐Navigator allows to efficiently analyze different aspects of NPs such as chemotype distribution, physicochemical properties, biological activity, and commercial availability of NPs.

The use of multiple structure representations to explore the chemical space of compound data sets and its impact to analyze structure‐activity relationships under the concept of activity landscapes has been shown and reviewed in the literature.^[56]^ In an early work, 2D and 3D representations were used to identify activity cliffs of a data set of 48 bicyclic guanidines with κ‐opioid receptor binding affinity. It was concluded that while some activity cliffs are dependent on the structure representation, there are activity cliffs that are consistent regardless of the representation explored, i. e. consensus activity cliffs.^[51]^

In order to generate efficient methods to quantify the diversity of large and ultra‐large chemical libraries and visualize their mutual relationships in chemical space, Dunn et al. developed Chemical Library Networks (CLNs) based on extended similarity indices.^[54]^ In this work, different CLNs of 19 chemical libraries used in drug discovery and NPs research were generated using MACCS keys (166‐bits), RDKit, and ECFP4 fingerprints. The analysis and comparison of the generated CLNs led to the conclusion that the extended Tanimoto index offers the best description of extended similarity in combination with RDKit fingerprints. In subsequent work, Flores‐Padilla et al. used CLNs and Constellation plots based on chemical core scaffolds to analyze 11 commercial libraries of different sizes focused on epigenetic targets (with 53443 compounds in total).^[3]^ The chemical space content and diversity analysis based on different descriptors helped to identify the most diverse synthetic‐focused screening libraries.

In the design of compound libraries, various representations of chemical space are also commonly used to assess the novelty, pharmaceutical properties, and molecular and shape diversity of the compounds generated. For example, to design combinatorial libraries it is common to analyze the chemical space based on physicochemical properties and compare it with sets of pharmaceutical relevance such as approved drugs. Structural novelty and diversity are also often visualized through chemical space based on molecular fingerprints. Other visualizations that are included in compound library design programs are the Principal Moments of Inertia plots. These graphs are mainly used in diversity‐oriented synthesis approaches to ensure the diversity of shapes of the designed compounds.^[57]^

Recently, Arús‐Pous et al. used different PCA plots of molecular quantum number fingerprints in compound library design to assess the quality of the training process in generative models.^[53]^ In that study, the obtained plots showed that when using recurrent neural networks to train models that sample chemical space, complex molecules with many rings and heteroatoms are more difficult to sample than molecules with fewer rings and more carbon atoms.

The encoding of fragments or building blocks, as well as experimental data on reactions in computer‐accessible formats, are opening new ways of representing chemical space. Integrating this information into the chemical space representation can facilitate the search for promising compounds in ultra‐large data collections and focus the synthesis of new compounds in relevant medicinal chemistry spaces.^[58]^ In another application of GTMs, Zabolotna et al. analyzed the diversity of more than 400,000 purchasable building blocks (PBBs) provided by eMolecules.^[55]^ Comparison of PBBs with synthons derived from ChEMBL fragmentation revealed that the internal diversity among members of the same class of synthons is significantly better for ChEMBL‐derived synthons, leaving room for the design and improvement of corresponding PBBs. Similarly, the existence of structurally equivalent synthons in ChEMBL can be used to search for alternative synthesis ways in situations where the same structural remainder can be provided by radically different reactivity BBs applicable in different synthetic routes.

**Figure 4:**
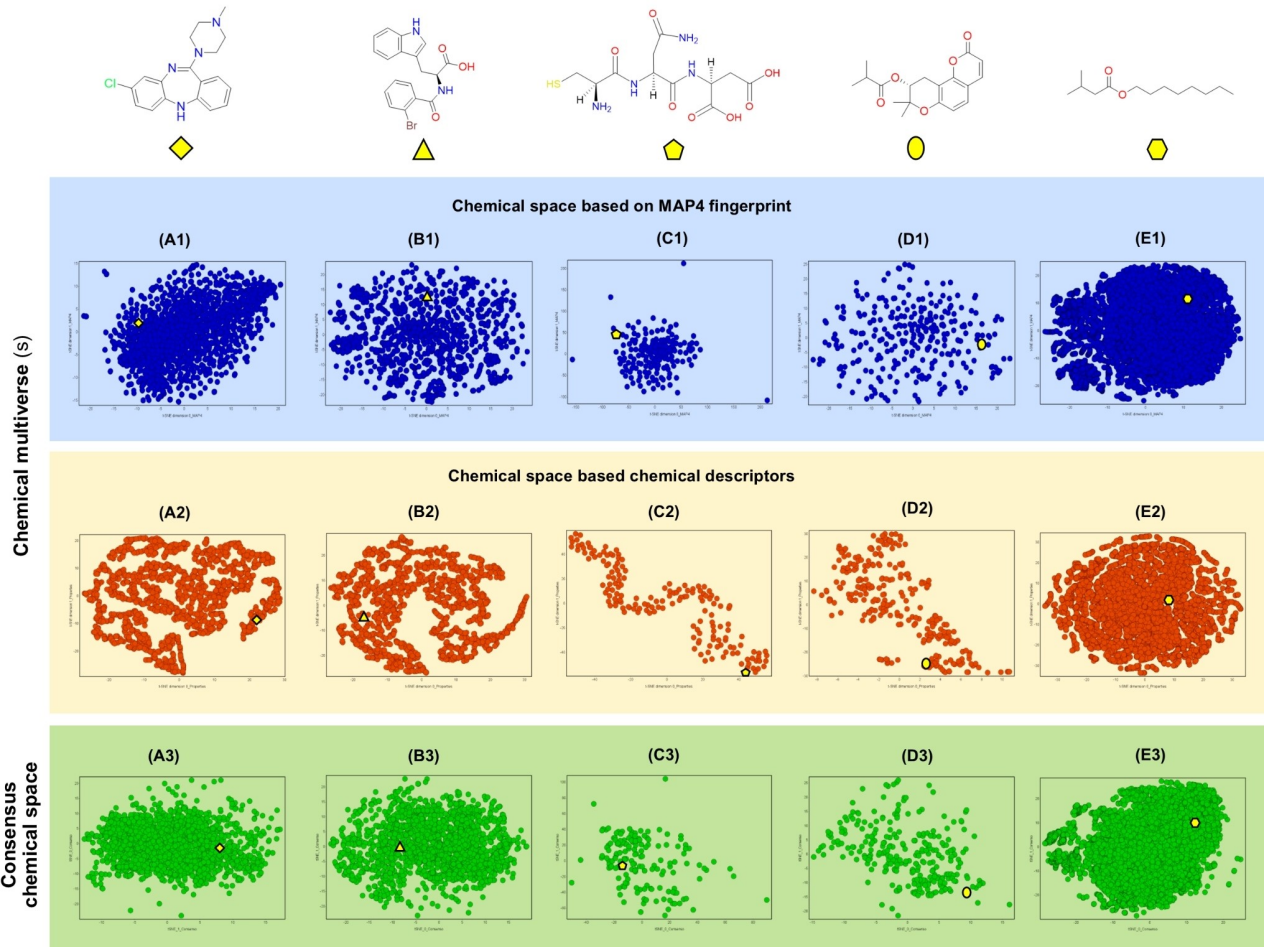
Example of the visual representation of chemical multiverse of four compound data sets: **A**) drug‐like (2,403 compounds); **B**) protein‐protein interaction inhibitors (2,227 compounds);^[65]^ **C**) anti‐MRSA peptides (165);^[59]^ **D**) natural products (285 compounds from BIOFACQUIM database);^[66]^ **E**) food chemicals (21,319 compounds from FooDB).^[67]^ The visual representations were obtained from the t‐SNE module implemented in KNIME. The chemical multiverse of each set is compared with a consensus representation of the chemical space obtained from averaging (i.e, average fusion rule) the coordinates of the data points. The upper part of this figure shows examples of representative chemical structures of each data set, each structure is represented by a yellow shape.

Figure 4 shows additional examples of visualization of chemical multiverses for different data sets: different nature of chemical structures and a varied number of molecules. Of note, the purpose of the examples in this figure showing data sets of varying chemical nature is to illustrate the concept of the chemical multiverse. Still, we do not attempt to make a direct comparison between the data sets or discuss their diversity. Figure 4 shows a 2D visualization of the chemical multiverse (given by two chemical spaces, each obtained with a different set of descriptors) and a representative consensus chemical space of five data sets from various sources: small organic drug‐like compounds, protein−protein interaction inhibitors, anti‐*Staphylococcus aureus* methicillin‐resistant (MRSA) peptides from the antimicrobial peptide database 3,^[59]^ natural products, and food chemicals. The coordinates for each graph in Figure 4 were created using the “tSNE module”^[14]^ of KNIME software (version 4.3.4).^[60]^ Figure 4A1–E1 (top part of the figure, in blue) shows a visualization of the chemical space based on the recently developed “MinHashed atom‐pair fingerprint up to a diameter of four bonds” (MAP4) fingerprint (2048 bits).^[61]^ Figure 4A2–E2 (middle of the figure, in orange) shows the visual representation of the chemical space using a different set of chemical descriptors: SlogP, TPSA, MW, Rotatable bonds, NumHBD, NumHBA, NumStereocenters, FractionCSP3, that were calculated with the RDKIt module implemented in KNIME.^[62]^ In this illustrative example, both chemical space representations in blue and orange of the five different data sets (A–E) shows a visualization of the chemical multiverse of the sets, in this case using t‐SNE (other approaches to visualize the chemical space or chemical multiverses can be employed, as described in Section 3). The chemical multiverses for each data set, e. g., comparing the pair of chemical spaces A1−A2, B1−B2, C1−C2, D1−D2, and E1−E2, clearly show the dependence of the chemical space on the structure representation, in these cases MAP4 fingerprints and chemical descriptors. This is particularly dramatic for data sets such as peptides and NPs.

For comparison, Figure 4A3–E3 (bottom part of the figure, in green) illustrates an example of a visualization of a consensus chemical space of the same five data sets. For this example, the consensus representation was generated by taking the average of the t‐SNE coordinates of each data set. Several other combinations of descriptors or fusion rules could be employed and finding “the best” one would not be straightforward. Of note, exploring and comparing different approaches to combine descriptors is beyond the scope of this review (in general) and the Figure 4 (in particular). As discussed in Section 3.1, the consensus representation of the chemical space would be directed to generating a “global” perception of the chemical universe, which is also valuable. To illustrate further the interdependence of data between the multiverse chemical spaces and consensus representation, the upper part of Figure 4 shows representative chemical structures of each data set. In general, the change in the relative position of each chemical structure in their chemical multiverse is condensed on the consensus representation of each one. However, the interpretation of the consensus chemical space becomes more complicated if one wants to associate the structure of property features that distinguish the compound data sets.

The interactive visualizations of chemical spaces were generated using DataWarrior software.[^63^, ^64^] The visualizations are available on Figshare at https://doi.org/10.6084/m9.figshare.20483958.

In Figures 4 alternative and more molecular representations could be used to illustrate the concept of chemical multiverses of the various data sets in addition to the published examples reviewed in Table 3. For illustration, only a few molecular representations were used.

The survey of published case studies (Table 3) shows the relevance of using several descriptors for various type of applications, namely structure‐activity relationships, diversity analysis, virtual screening, compound library design, visual representation of the chemical space, and compound selection. The examples in Figure 4 are intended to further illustrate that chemical multiverses can be applied to virtually any type of molecular structure such as small molecules as drug candidates, NPs, peptides, inhibitors of protein−protein interactions, peptides, and food chemicals, that have distinct structural features.

## 5 Summary and Outlook

The number and type of chemical structures relevant to drug discovery and other related applications are dramatically increasing. Similarly, the number and type of experimental or calculated descriptors also augment. The continued increase in the number of compounds and descriptors encourages novel ways to interact with the chemical space beyond the traditional medicinally relevant chemical space built based on drug‐ or lead‐like properties of the pharmaceutical interest and the conventional organic small‐molecules. Herein, we introduce the term *chemical multiverse* as a group of chemical spaces, each one defined by a given set of descriptors. By its own nature, a chemical multiverse provides more information about a single chemical space defined by a specific molecular representation. In this review, we have shown that the use of multiple descriptors to study the chemical space has been implemented in several studies as a single type of descriptor is not enough to capture all the required features for structure‐activity relationships, or other applications such as diversity analysis, virtual screening, compound library design, visual representation of the chemical space, and compound selection. A “chemical megaverse” is the collection of chemical multiverses, and this would be given by the several different groups of descriptors that can be used to define a chemical space.

For some specific compound data sets, for instance, metal‐containing molecules and other types of complex molecules, it remains to determine the chemical multiverses consistently: very much like in cosmology, to explore uncharted regions of the chemical space. Also, it remains to explore how to move from one universe to another (aka, navigating between chemical spaces or *navigating the chemical multiverse* of a compound data set), similar to physics or astronomy, but we can find a point where each multiverse connects or overlaps, and we can observe these points in the visualizations of chemical spaces.

Beyond this review paper that surveys studies that use different descriptors for various drug discovery applications, it remains to compare, in a research study, different ways to combine descriptors and, in general, generate consensus representations of chemical spaces.

As illustrated in this manuscript, same as the chemical space concept, the chemical multiverse and chemical megaverse have applications in several research areas, including drug discovery and beyond.

## Abbreviations

2D, two‐dimensional, 3D, three‐dimensional; CLNs, Chemical Library Networks; ECFP, extended connectivity fingerprint; FDA, Food and Drug Administration; GTM, Generative Topographic Mapping; HIV, human immunodeficiency virus; MAP4, MinHashed atom‐pair fingerprint up to a diameter of four bonds; MRSA, anti‐*Staphylococcus aureus* methicillin‐resistant; NPs, natural products; PCA, principal component analysis; Rule of five, favorable pharmacokinetic properties in terms of absorption and distribution, described by molecular weight (MW)≤500, partition coefficient octanol/water (SlogP)≤5, hydrogen bond acceptors (HBA)≤10, hydrogen bond donors (HBD) ≤5; SOM, self‐organized map; SAR, structure‐activity relationships; t‐SNE, t‐distributed stochastic neighbor embedding.

## Author Contribution Statement

All authors have contributed equally to the present manuscript.

## Conflict of interest

None declared.

## Biographical Information

*José L. Medina‐Franco received his Ph.D. degree from the National Autonomous University of Mexico (UNAM). He was a postdoctoral fellow at the University of Arizona and joined the Torrey Pines Institute for Molecular Studies in Florida in 2007. In 2013, he moved to the Mayo Clinic and later joined UNAM as Full Time Research Professor. He currently leads the DIFACQUIM research group. In 2017 he was named Fellow of the Royal Society of Chemistry. His research interests include the development and application of chemoinformatics and molecular modeling methods for bioactive compounds with an emphasis on drug discovery*.

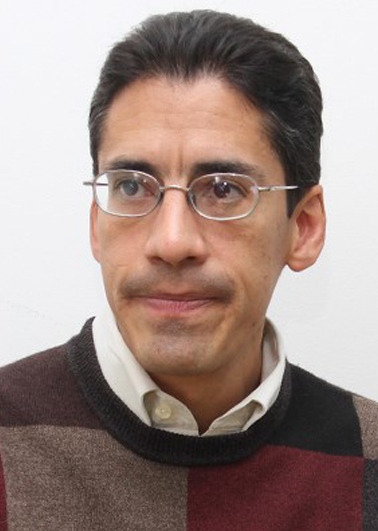

## Biographical Information

*Ana Luisa Chávez‐Hernández received her BSc degree in Food Engineering (2016) from the Autonomy Metropolitan University (UAM) and her Master's degree in Chemical Science (2019) from the National Autonomous University of Mexico (UNAM). Currently, she is a Ph.D. student in Chemistry Science under the supervision of Professor José Luis Medina‐Franco; her research focuses on the development of libraries from natural products for de novo drug design*.

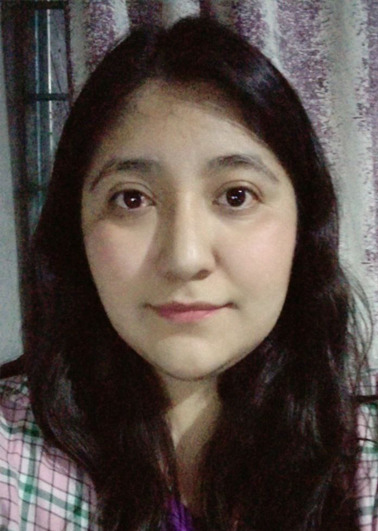

## Biographical Information

*Edgar López‐López received is B.S. degree in Clinical Chemistry from the University of Veracruz, Mexico, in 2019, and an M.Sc. degree in pharmacology from the Center for Research and Advanced Studies of the National Polytechnic Institute (CINVESTAV), Mexico, in 2021. He is currently a Ph.D. student, and his research interest includes the design, synthesis, and biological evaluation of anticancer and antiparasitic drugs*.

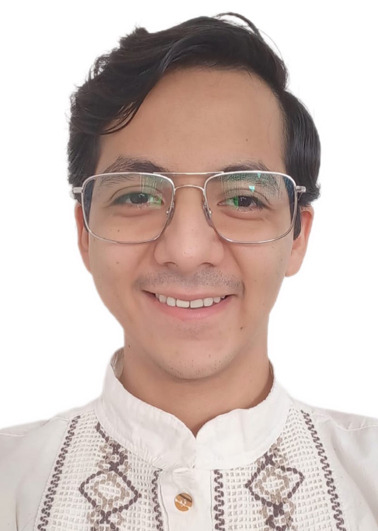

## Biographical Information

*Fernanda I. Saldivar‐Gonzalez received her BSc degree in Chemistry Pharmacy and Biology (2017) from the National Autonomous University of Mexico (UNAM). She received the Master′s degree in Chemical Sciences in 2019 under the supervision of Professor José Luis Medina‐Franco, after spending a research period in the group of Prof. Andrea Trabocchi at the University of Florence, Italy. She is currently a Ph.D. student in Chemistry in the area of pharmacy where she develops her project focused on the design of virtual chemical libraries of antidiabetic compounds*.

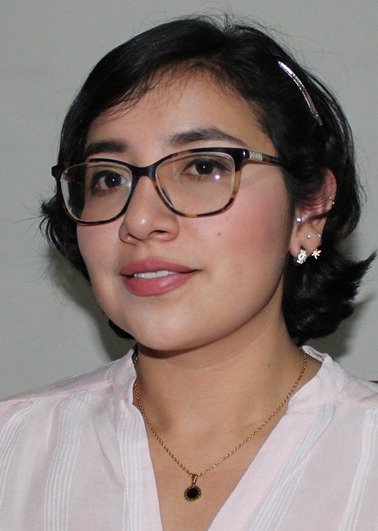

## Data Availability

The data that supports the findings of this study are freely available on Figshare at https://doi.org/10.6084/m9.figshare.19768174.
